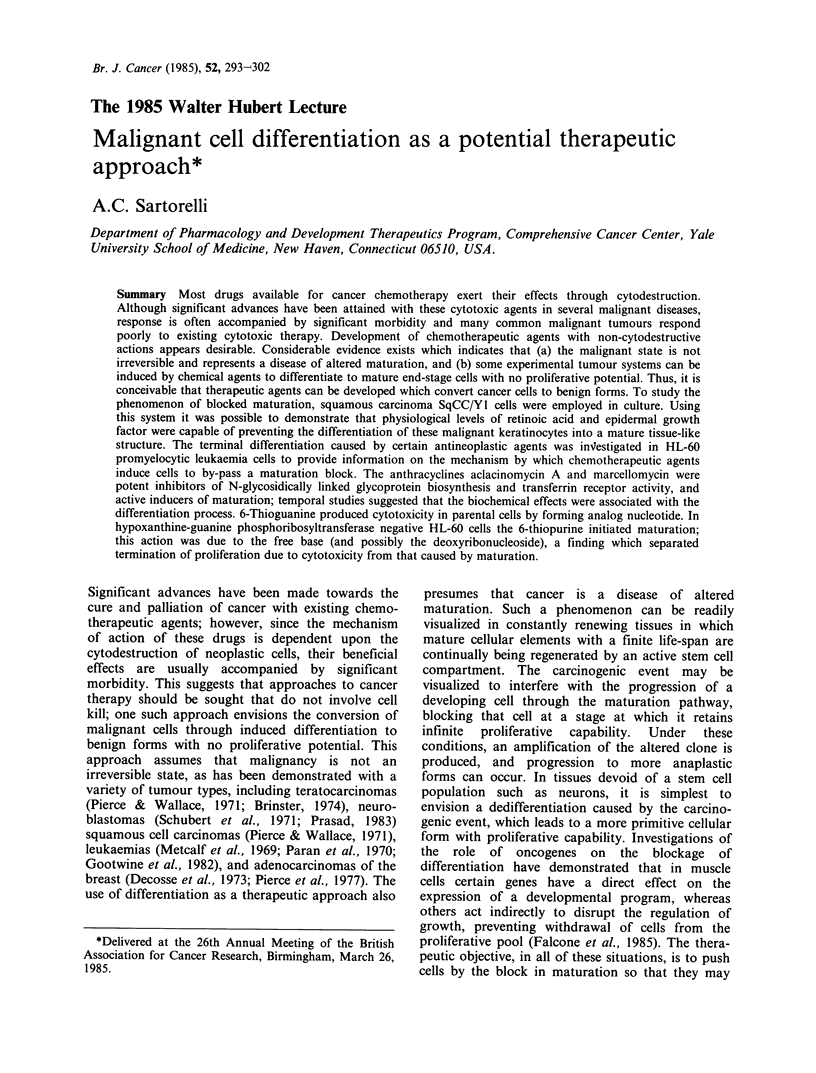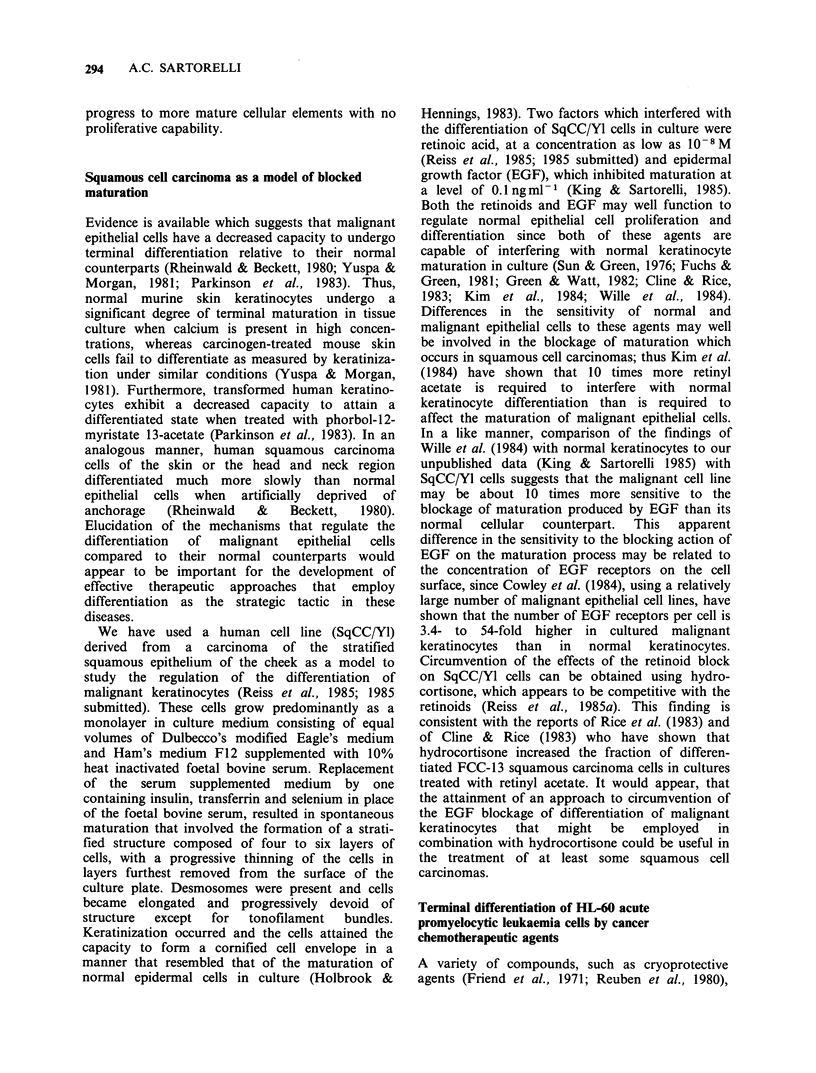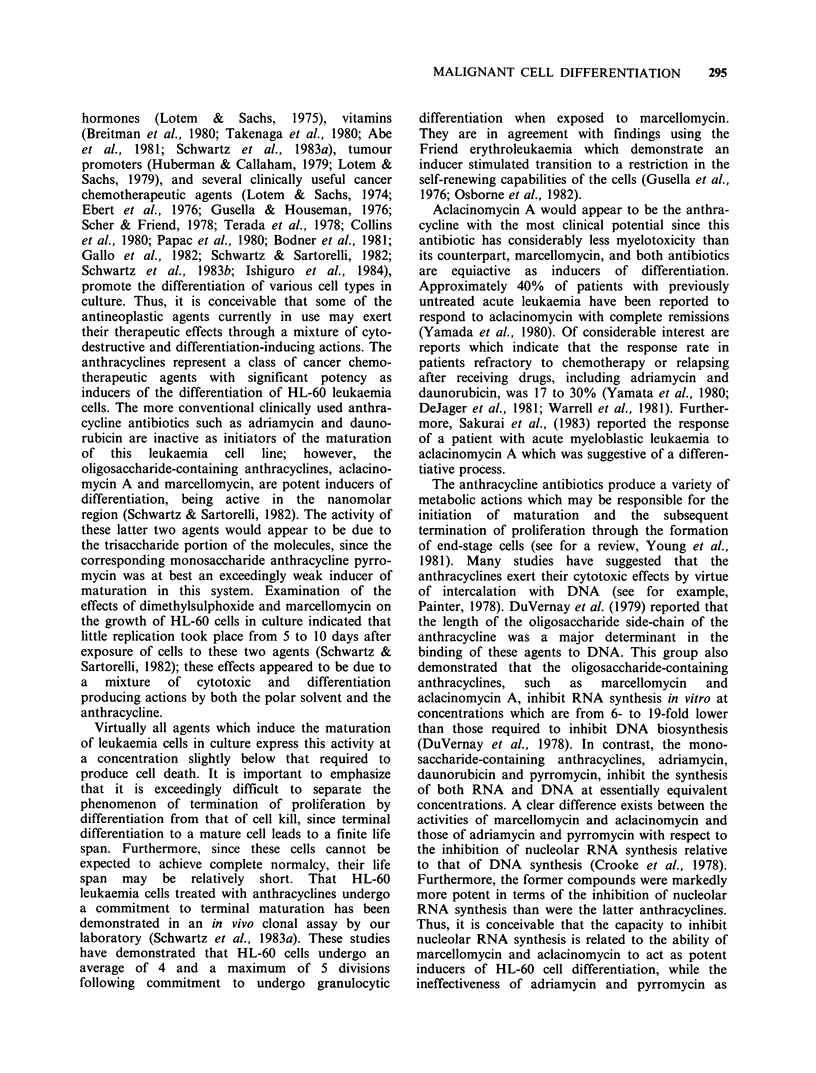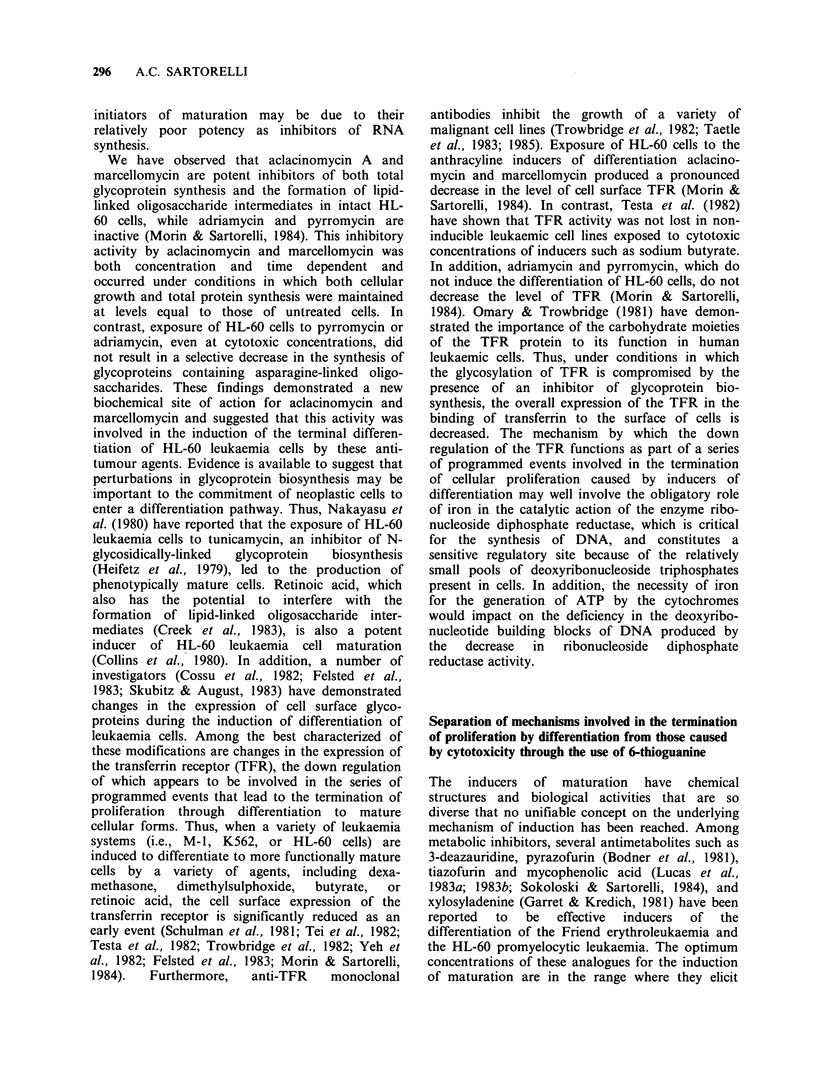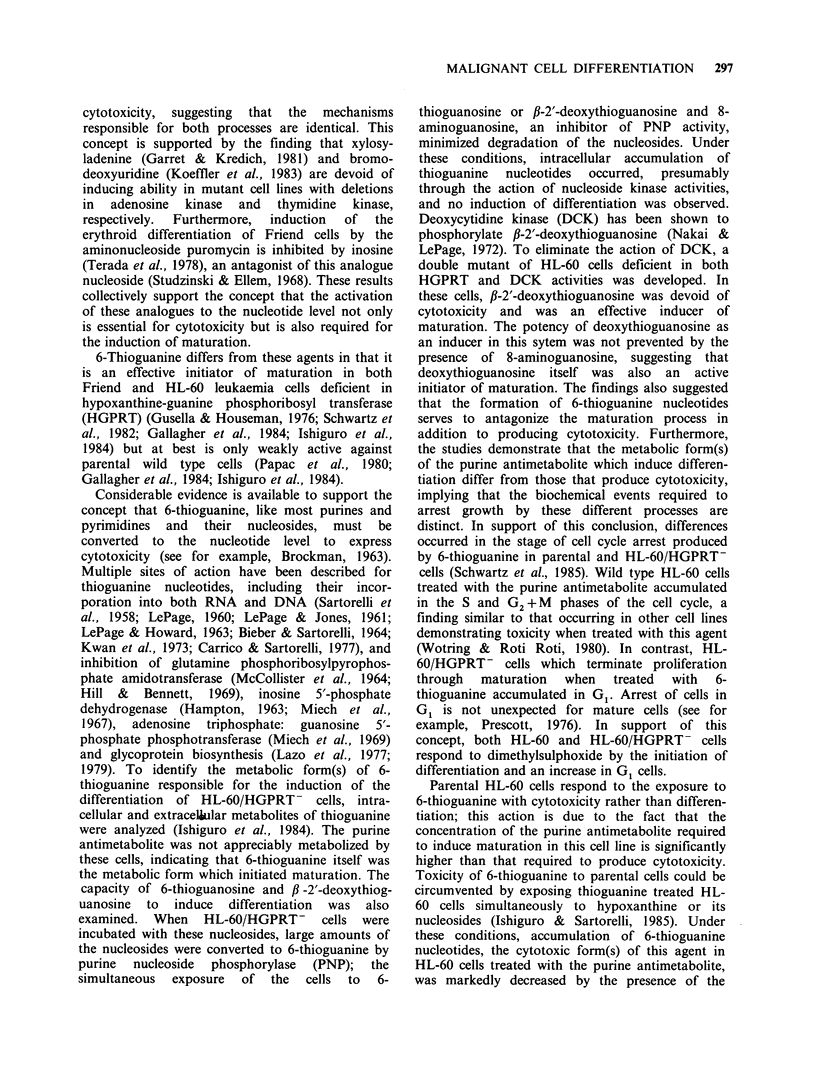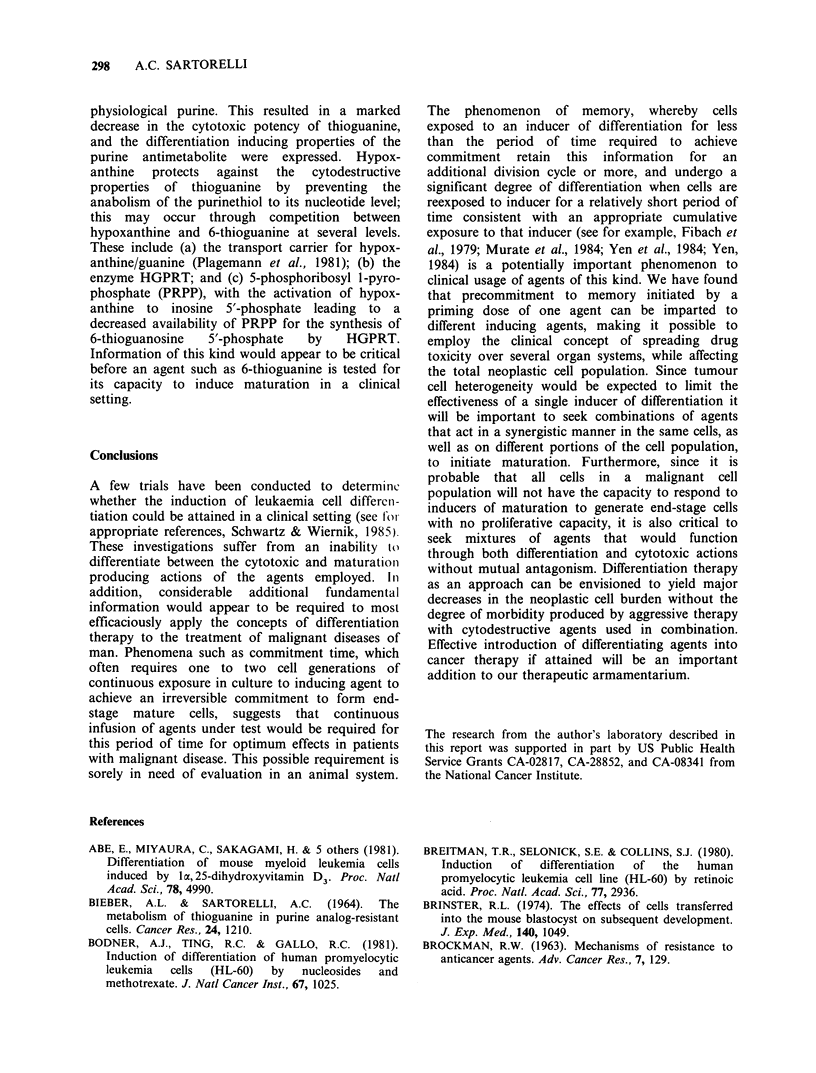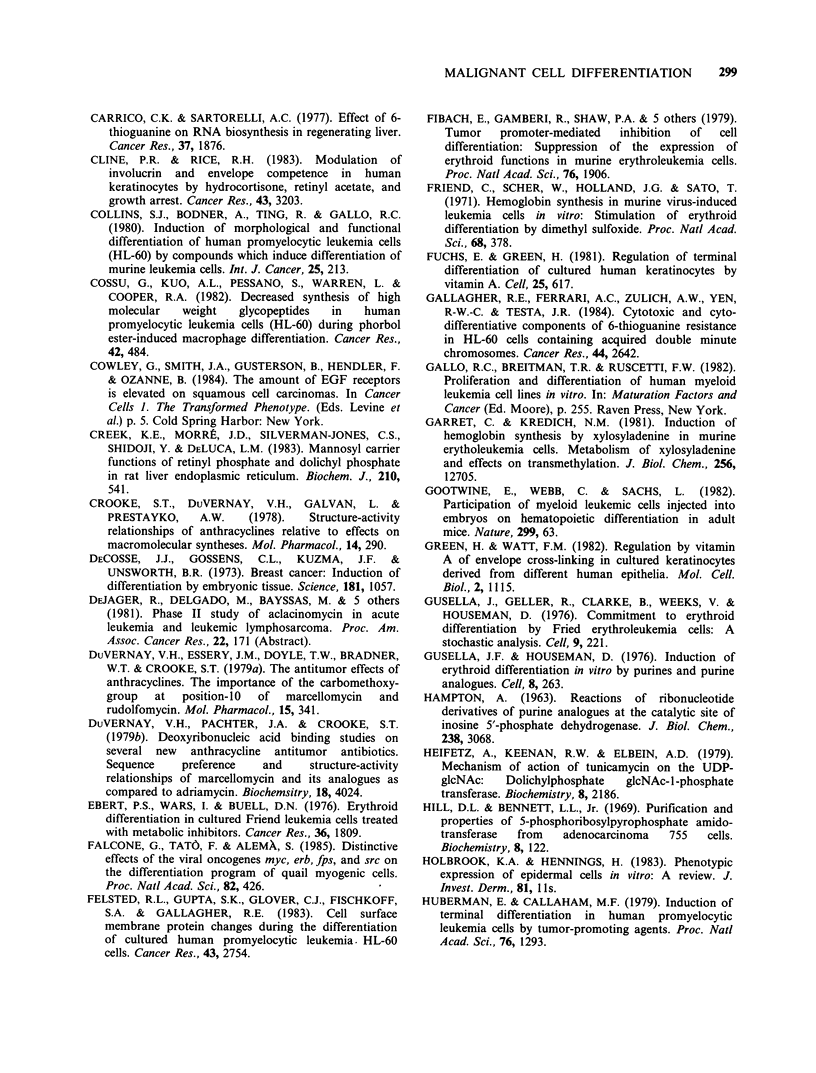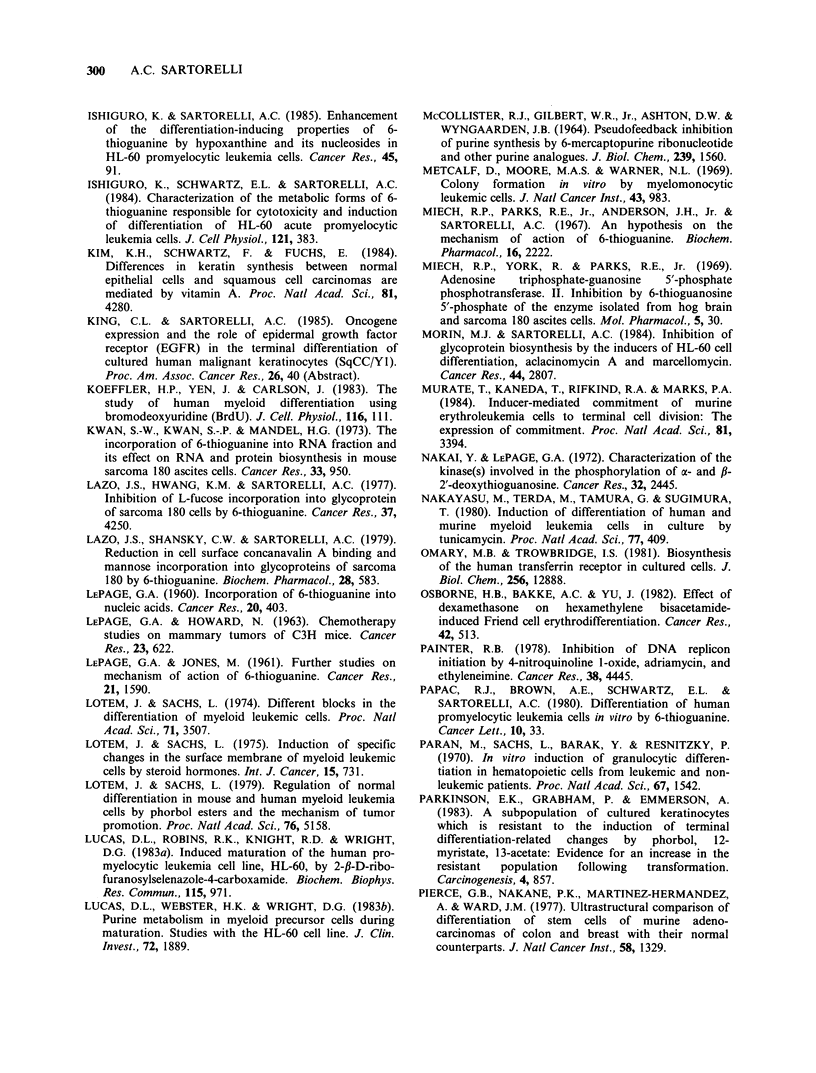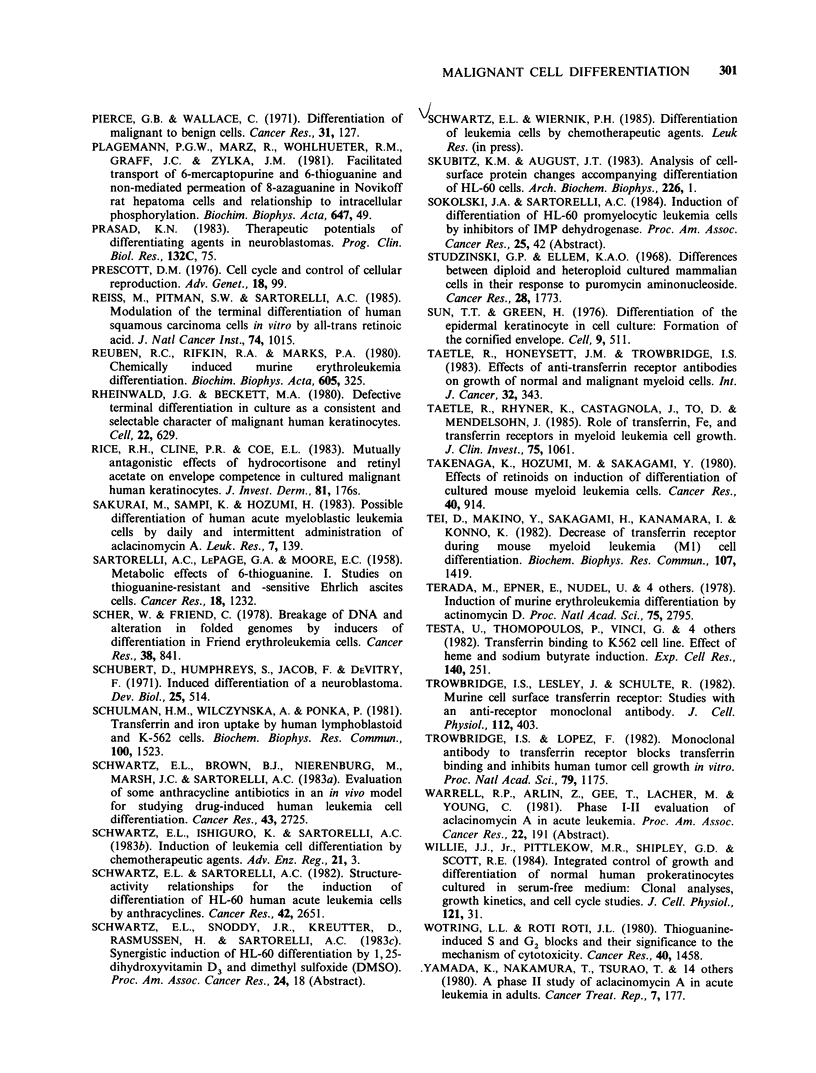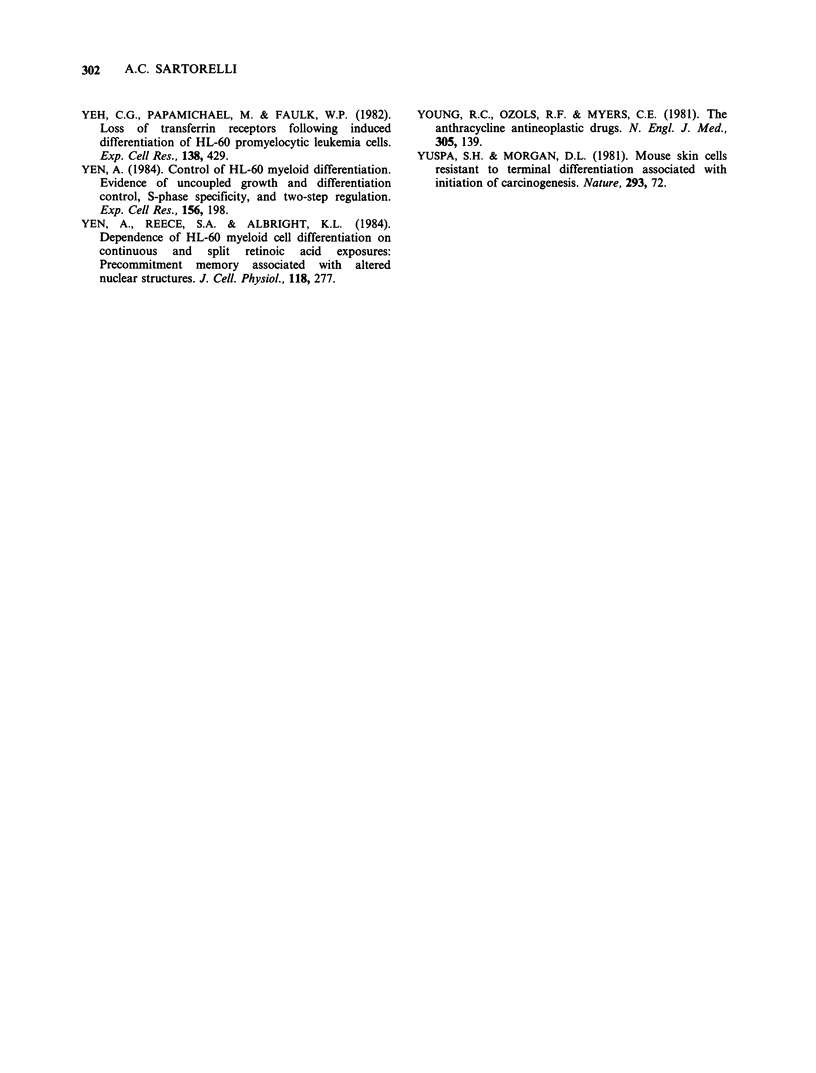# The 1985 Walter Hubert lecture. Malignant cell differentiation as a potential therapeutic approach.

**DOI:** 10.1038/bjc.1985.193

**Published:** 1985-09

**Authors:** A. C. Sartorelli

## Abstract

Most drugs available for cancer chemotherapy exert their effects through cytodestruction. Although significant advances have been attained with these cytotoxic agents in several malignant diseases, response is often accompanied by significant morbidity and many common malignant tumours respond poorly to existing cytotoxic therapy. Development of chemotherapeutic agents with non-cytodestructive actions appears desirable. Considerable evidence exists which indicates that (a) the malignant state is not irreversible and represents a disease of altered maturation, and (b) some experimental tumour systems can be induced by chemical agents to differentiate to mature end-stage cells with no proliferative potential. Thus, it is conceivable that therapeutic agents can be developed which convert cancer cells to benign forms. To study the phenomenon of blocked maturation, squamous carcinoma SqCC/Y1 cells were employed in culture. Using this system it was possible to demonstrate that physiological levels of retinoic acid and epidermal growth factor were capable of preventing the differentiation of these malignant keratinocytes into a mature tissue-like structure. The terminal differentiation caused by certain antineoplastic agents was investigated in HL-60 promyelocytic leukaemia cells to provide information on the mechanism by which chemotherapeutic agents induce cells to by-pass a maturation block. The anthracyclines aclacinomycin A and marcellomycin were potent inhibitors of N-glycosidically linked glycoprotein biosynthesis and transferrin receptor activity, and active inducers of maturation; temporal studies suggested that the biochemical effects were associated with the differentiation process. 6-Thioguanine produced cytotoxicity in parental cells by forming analog nucleotide. In hypoxanthine-guanine phosphoribosyltransferase negative HL-60 cells the 6-thiopurine initiated maturation; this action was due to the free base (and possibly the deoxyribonucleoside), a finding which separated termination of proliferation due to cytotoxicity from that caused by maturation.


*Br. J. Cancer* (1985), **52**, 293–302

**The 1985 Walter Hubert Lecture**

### Malignant cell differentiation as a potential therapeutic approach*

A.C. Sartorelli

*Department of Pharmacology and Development Therapeutics Program, Comprehensive Cancer Center, Yale University School of Medicine, New Haven, Connecticut 06510, USA.*

**Summary** Most drugs available for cancer chemotherapy exert their effects through cytodestruction. Although significant advances have been attained with these cytotoxic agents in several malignant diseases, response is often accompanied by significant morbidity and many common malignant tumours respond poorly to existing cytotoxic therapy. Development of chemotherapeutic agents with non-cytodestructive actions appears desirable. Considerable evidence exists which indicates that (a) the malignant state is not irreversible and represents a disease of altered maturation, and (b) some experimental tumour systems can be induced by chemical agents to differentiate to mature end-stage cells with no proliferative potential. Thus, it is conceivable that therapeutic agents can be developed which convert cancer cells to benign forms. To study the phenomenon of blocked maturation, squamous carcinoma SqCC/Y1 cells were employed in culture. Using this system it was possible to demonstrate that physiological levels of retinoic acid and epidermal growth factor were capable of preventing the differentiation of these malignant keratinocytes into a mature tissue-like structure. The terminal differentiation caused by certain antineoplastic agents was investigated in HL-60 promyelocytic leukaemia cells to provide information on the mechanism by which chemotherapeutic agents induce cells to by-pass a maturation block. The anthracyclines aclacinomycin A and marcellomycin were potent inhibitors of N-glycosidically linked glycoprotein biosynthesis and transferrin receptor activity, and active inducers of maturation; temporal studies suggested that the biochemical effects were associated with the differentiation process. 6-Thioguanine produced cytotoxicity in parental cells by forming analog nucleotide. In hypoxanthine-guanine phosphoribosyltransferase negative HL-60 cells the 6-thiopurine initiated maturation; this action was due to the free base (and possibly the deoxyribonucleoside), a finding which separated termination of proliferation due to cytotoxicity from that caused by maturation.

Significant advances have been made towards the cure and palliation of cancer with existing chemotherapeutic agents; however, since the mechanism of action of these drugs is dependent upon the cytodestruction of neoplastic cells, their beneficial effects are usually accompanied by significant morbidity. This suggests that approaches to cancer therapy should be sought that do not involve cell kill; one such approach envisions the conversion of malignant cells through induced differentiation to benign forms with no proliferative potential. This approach assumes that malignancy is not an irreversible state, as has been demonstrated with a variety of tumour types, including teratocarcinomas (Pierce & Wallace, 1971; Brinster, 1974), neuroblastomas (Schubert *et al.*, 1971; Prasad, 1983) squamous cell carcinomas (Pierce & Wallace, 1971), leukaemias (Metcalf *et al.*, 1969; Paran *et al.*, 1970; Gootwine *et al.*, 1982), and adenocarcinomas of the breast (Decosse *et al.*, 1973; Pierce *et al.*, 1977). The use of differentiation as a therapeutic approach also presumes that cancer is a disease of altered maturation. Such a phenomenon can be readily visualized in constantly renewing tissues in which mature cellular elements with a finite life-span are continually being regenerated by an active stem cell compartment. The carcinogenic event may be visualized to interfere with the progression of a developing cell through the maturation pathway, blocking that cell at a stage at which it retains infinite proliferative capability. Under these conditions, an amplification of the altered clone is produced, and progression to more anaplastic forms can occur. In tissues devoid of a stem cell population such as neurons, it is simplest to envision a dedifferentiation caused by the carcinogenic event, which leads to a more primitive cellular form with proliferative capability. Investigations of the role of oncogenes on the blockage of differentiation have demonstrated that in muscle cells certain genes have a direct effect on the expression of a developmental program, whereas others act indirectly to disrupt the regulation of growth, preventing withdrawal of cells from the proliferative pool (Falcone *et al.*, 1985). The therapeutic objective, in all of these situations, is to push cells by the block in maturation so that they may

*Delivered at the 26th Annual Meeting of the British Association for Cancer Research, Birmingham, March 26, 1985.

progress to more mature cellular elements with no proliferative capability.

### Squamous cell carcinoma as a model of blocked maturation

Evidence is available which suggests that malignant epithelial cells have a decreased capacity to undergo terminal differentiation relative to their normal counterparts (Rheinwald & Beckett, 1980; Yuspa & Morgan, 1981; Parkinson *et al.,* 1983). Thus, normal murine skin keratinocytes undergo a significant degree of terminal maturation in tissue culture when calcium is present in high concentrations, whereas carcinogen-treated mouse skin cells fail to differentiate as measured by keratinization under similar conditions (Yuspa & Morgan, 1981). Furthermore, transformed human keratinocytes exhibit a decreased capacity to attain a differentiated state when treated with phorbol-12-myristate 13-acetate (Parkinson *et al.,* 1983). In an analogous manner, human squamous carcinoma cells of the skin or the head and neck region differentiated much more slowly than normal epithelial cells when artificially deprived of anchorage (Rheinwald & Beckett, 1980). Elucidation of the mechanisms that regulate the differentiation of malignant epithelial cells compared to their normal counterparts would appear to be important for the development of effective therapeutic approaches that employ differentiation as the strategic tactic in these diseases.

We have used a human cell line (SqCC/Yl) derived from a carcinoma of the stratified squamous epithelium of the cheek as a model to study the regulation of the differentiation of malignant keratinocytes (Reiss *et al.,* 1985; 1985 submitted). These cells grow predominantly as a monolayer in culture medium consisting of equal volumes of Dulbecco's modified Eagle's medium and Ham's medium F12 supplemented with 10% heat inactivated foetal bovine serum. Replacement of the serum supplemented medium by one containing insulin, transferrin and selenium in place of the foetal bovine serum, resulted in spontaneous maturation that involved the formation of a stratified structure composed of four to six layers of cells, with a progressive thinning of the cells in layers furthest removed from the surface of the culture plate. Desmosomes were present and cells became elongated and progressively devoid of structure except for tonofilament bundles. Keratinization occurred and the cells attained the capacity to form a cornified cell envelope in a manner that resembled that of the maturation of normal epidermal cells in culture (Holbrook & Hennings, 1983). Two factors which interfered with the differentiation of SqCC/Yl cells in culture were retinoic acid, at a concentration as low as $10^{-8}$ M (Reiss *et al.,* 1985; 1985 submitted) and epidermal growth factor (EGF), which inhibited maturation at a level of 0.1 ng ml$^{-1}$ (King & Sartorelli, 1985). Both the retinoids and EGF may well function to regulate normal epithelial cell proliferation and differentiation since both of these agents are capable of interfering with normal keratinocyte maturation in culture (Sun & Green, 1976; Fuchs & Green, 1981; Green & Watt, 1982; Cline & Rice, 1983; Kim *et al.,* 1984; Wille *et al.,* 1984). Differences in the sensitivity of normal and malignant epithelial cells to these agents may well be involved in the blockage of maturation which occurs in squamous cell carcinomas; thus Kim *et al.* (1984) have shown that 10 times more retinyl acetate is required to interfere with normal keratinocyte differentiation than is required to affect the maturation of malignant epithelial cells. In a like manner, comparison of the findings of Wille *et al.* (1984) with normal keratinocytes to our unpublished data (King & Sartorelli 1985) with SqCC/Yl cells suggests that the malignant cell line may be about 10 times more sensitive to the blockage of maturation produced by EGF than its normal cellular counterpart. This apparent difference in the sensitivity to the blocking action of EGF on the maturation process may be related to the concentration of EGF receptors on the cell surface, since Cowley *et al.* (1984), using a relatively large number of malignant epithelial cell lines, have shown that the number of EGF receptors per cell is 3.4- to 54-fold higher in cultured malignant keratinocytes than in normal keratinocytes. Circumvention of the effects of the retinoid block on SqCC/Yl cells can be obtained using hydrocortisone, which appears to be competitive with the retinoids (Reiss *et al.,* 1985*a*). This finding is consistent with the reports of Rice *et al.* (1983) and of Cline & Rice (1983) who have shown that hydrocortisone increased the fraction of differentiated FCC-13 squamous carcinoma cells in cultures treated with retinyl acetate. It would appear, that the attainment of an approach to circumvention of the EGF blockage of differentiation of malignant keratinocytes that might be employed in combination with hydrocortisone could be useful in the treatment of at least some squamous cell carcinomas.

### Terminal differentiation of HL-60 acute promyelocytic leukaemia cells by cancer chemotherapeutic agents

A variety of compounds, such as cryoprotective agents (Friend *et al.,* 1971; Reuben *et al.,* 1980), hormones (Lotem & Sachs, 1975), vitamins (Breitman *et al.*, 1980; Takenaga *et al.*, 1980; Abe *et al.*, 1981; Schwartz *et al.*, 1983*a*), tumour promoters (Huberman & Callaham, 1979; Lotem & Sachs, 1979), and several clinically useful cancer chemotherapeutic agents (Lotem & Sachs, 1974; Ebert *et al.*, 1976; Gusella & Houseman, 1976; Scher & Friend, 1978; Terada *et al.*, 1978; Collins *et al.*, 1980; Papac *et al.*, 1980; Bodner *et al.*, 1981; Gallo *et al.*, 1982; Schwartz & Sartorelli, 1982; Schwartz *et al.*, 1983*b*; Ishiguro *et al.*, 1984), promote the differentiation of various cell types in culture. Thus, it is conceivable that some of the antineoplastic agents currently in use may exert their therapeutic effects through a mixture of cytodestructive and differentiation-inducing actions. The anthracyclines represent a class of cancer chemotherapeutic agents with significant potency as inducers of the differentiation of HL-60 leukaemia cells. The more conventional clinically used anthracycline antibiotics such as adriamycin and daunorubicin are inactive as initiators of the maturation of this leukaemia cell line; however, the oligosaccharide-containing anthracyclines, aclacinomycin A and marcellomycin, are potent inducers of differentiation, being active in the nanomolar region (Schwartz & Sartorelli, 1982). The activity of these latter two agents would appear to be due to the trisaccharide portion of the molecules, since the corresponding monosaccharide anthracycline pyrromycin was at best an exceedingly weak inducer of maturation in this system. Examination of the effects of dimethylsulphoxide and marcellomycin on the growth of HL-60 cells in culture indicated that little replication took place from 5 to 10 days after exposure of cells to these two agents (Schwartz & Sartorelli, 1982); these effects appeared to be due to a mixture of cytotoxic and differentiation producing actions by both the polar solvent and the anthracycline.

Virtually all agents which induce the maturation of leukaemia cells in culture express this activity at a concentration slightly below that required to produce cell death. It is important to emphasize that it is exceedingly difficult to separate the phenomenon of termination of proliferation by differentiation from that of cell kill, since terminal differentiation to a mature cell leads to a finite life span. Furthermore, since these cells cannot be expected to achieve complete normalcy, their life span may be relatively short. That HL-60 leukaemia cells treated with anthracyclines undergo a commitment to terminal maturation has been demonstrated in an *in vivo* clonal assay by our laboratory (Schwartz *et al.*, 1983*a*). These studies have demonstrated that HL-60 cells undergo an average of 4 and a maximum of 5 divisions following commitment to undergo granulocytic differentiation when exposed to marcellomycin. They are in agreement with findings using the Friend erythroleukaemia which demonstrate an inducer stimulated transition to a restriction in the self-renewing capabilities of the cells (Gusella *et al.*, 1976; Osborne *et al.*, 1982).

Aclacinomycin A would appear to be the anthracycline with the most clinical potential since this antibiotic has considerably less myelotoxicity than its counterpart, marcellomycin, and both antibiotics are equiactive as inducers of differentiation. Approximately 40% of patients with previously untreated acute leukaemia have been reported to respond to aclacinomycin with complete remissions (Yamada *et al.*, 1980). Of considerable interest are reports which indicate that the response rate in patients refractory to chemotherapy or relapsing after receiving drugs, including adriamycin and daunorubicin, was 17 to 30% (Yamata *et al.*, 1980; DeJager *et al.*, 1981; Warrell *et al.*, 1981). Furthermore, Sakurai *et al.*, (1983) reported the response of a patient with acute myeloblastic leukaemia to aclacinomycin A which was suggestive of a differentiative process.

The anthracycline antibiotics produce a variety of metabolic actions which may be responsible for the initiation of maturation and the subsequent termination of proliferation through the formation of end-stage cells (see for a review, Young *et al.*, 1981). Many studies have suggested that the anthracyclines exert their cytotoxic effects by virtue of intercalation with DNA (see for example, Painter, 1978). DuVernay *et al.* (1979) reported that the length of the oligosaccharide side-chain of the anthracycline was a major determinant in the binding of these agents to DNA. This group also demonstrated that the oligosaccharide-containing anthracyclines, such as marcellomycin and aclacinomycin A, inhibit RNA synthesis *in vitro* at concentrations which are from 6- to 19-fold lower than those required to inhibit DNA biosynthesis (DuVernay *et al.*, 1978). In contrast, the monosaccharide-containing anthracyclines, adriamycin, daunorubicin and pyrromycin, inhibit the synthesis of both RNA and DNA at essentially equivalent concentrations. A clear difference exists between the activities of marcellomycin and aclacinomycin and those of adriamycin and pyrromycin with respect to the inhibition of nucleolar RNA synthesis relative to that of DNA synthesis (Crooke *et al.*, 1978). Furthermore, the former compounds were markedly more potent in terms of the inhibition of nucleolar RNA synthesis than were the latter anthracyclines. Thus, it is conceivable that the capacity to inhibit nucleolar RNA synthesis is related to the ability of marcellomycin and aclacinomycin to act as potent inducers of HL-60 cell differentiation, while the ineffectiveness of adriamycin and pyrromycin as initiators of maturation may be due to their relatively poor potency as inhibitors of RNA synthesis.

We have observed that aclacinomycin A and marcellomycin are potent inhibitors of both total glycoprotein synthesis and the formation of lipid-linked oligosaccharide intermediates in intact HL-60 cells, while adriamycin and pyrromycin are inactive (Morin & Sartorelli, 1984). This inhibitory activity by aclacinomycin and marcellomycin was both concentration and time dependent and occurred under conditions in which both cellular growth and total protein synthesis were maintained at levels equal to those of untreated cells. In contrast, exposure of HL-60 cells to pyrromycin or adriamycin, even at cytotoxic concentrations, did not result in a selective decrease in the synthesis of glycoproteins containing asparagine-linked oligosaccharides. These findings demonstrated a new biochemical site of action for aclacinomycin and marcellomycin and suggested that this activity was involved in the induction of the terminal differentiation of HL-60 leukaemia cells by these antitumour agents. Evidence is available to suggest that perturbations in glycoprotein biosynthesis may be important to the commitment of neoplastic cells to enter a differentiation pathway. Thus, Nakayasu *et al.* (1980) have reported that the exposure of HL-60 leukaemia cells to tunicamycin, an inhibitor of N-glycosidically-linked glycoprotein biosynthesis (Heifetz *et al.*, 1979), led to the production of phenotypically mature cells. Retinoic acid, which also has the potential to interfere with the formation of lipid-linked oligosaccharide intermediates (Creek *et al.*, 1983), is also a potent inducer of HL-60 leukaemia cell maturation (Collins *et al.*, 1980). In addition, a number of investigators (Cossu *et al.*, 1982; Felsted *et al.*, 1983; Skubitz & August, 1983) have demonstrated changes in the expression of cell surface glycoproteins during the induction of differentiation of leukaemia cells. Among the best characterized of these modifications are changes in the expression of the transferrin receptor (TFR), the down regulation of which appears to be involved in the series of programmed events that lead to the termination of proliferation through differentiation to mature cellular forms. Thus, when a variety of leukaemia systems (i.e., M-1, K562, or HL-60 cells) are induced to differentiate to more functionally mature cells by a variety of agents, including dexamethasone, dimethylsulphoxide, butyrate, or retinoic acid, the cell surface expression of the transferrin receptor is significantly reduced as an early event (Schulman *et al.*, 1981; Tei *et al.*, 1982; Testa *et al.*, 1982; Trowbridge *et al.*, 1982; Yeh *et al.*, 1982; Felsted *et al.*, 1983; Morin & Sartorelli, 1984). Furthermore, anti-TFR monoclonal antibodies inhibit the growth of a variety of malignant cell lines (Trowbridge *et al.*, 1982; Taetle *et al.*, 1983; 1985). Exposure of HL-60 cells to the anthracyline inducers of differentiation aclacinomycin and marcellomycin produced a pronounced decrease in the level of cell surface TFR (Morin & Sartorelli, 1984). In contrast, Testa *et al.* (1982) have shown that TFR activity was not lost in non-inducible leukaemic cell lines exposed to cytotoxic concentrations of inducers such as sodium butyrate. In addition, adriamycin and pyrromycin, which do not induce the differentiation of HL-60 cells, do not decrease the level of TFR (Morin & Sartorelli, 1984). Omary & Trowbridge (1981) have demonstrated the importance of the carbohydrate moieties of the TFR protein to its function in human leukaemic cells. Thus, under conditions in which the glycosylation of TFR is compromised by the presence of an inhibitor of glycoprotein biosynthesis, the overall expression of the TFR in the binding of transferrin to the surface of cells is decreased. The mechanism by which the down regulation of the TFR functions as part of a series of programmed events involved in the termination of cellular proliferation caused by inducers of differentiation may well involve the obligatory role of iron in the catalytic action of the enzyme ribonucleoside diphosphate reductase, which is critical for the synthesis of DNA, and constitutes a sensitive regulatory site because of the relatively small pools of deoxyribonucleoside triphosphates present in cells. In addition, the necessity of iron for the generation of ATP by the cytochromes would impact on the deficiency in the deoxyribonucleotide building blocks of DNA produced by the decrease in ribonucleoside diphosphate reductase activity.

### Separation of mechanisms involved in the termination of proliferation by differentiation from those caused by cytotoxicity through the use of 6-thioguanine

The inducers of maturation have chemical structures and biological activities that are so diverse that no unifiable concept on the underlying mechanism of induction has been reached. Among metabolic inhibitors, several antimetabolites such as 3-deazauridine, pyrazofurin (Bodner *et al.*, 1981), tiazofurin and mycophenolic acid (Lucas *et al.*, 1983*a*; 1983*b*; Sokoloski & Sartorelli, 1984), and xylosyladenine (Garret & Kredich, 1981) have been reported to be effective inducers of the differentiation of the Friend erythroleukaemia and the HL-60 promyelocytic leukaemia. The optimum concentrations of these analogues for the induction of maturation are in the range where they elicit cytotoxicity, suggesting that the mechanisms responsible for both processes are identical. This concept is supported by the finding that xylosyladenine (Garret & Kredich, 1981) and bromodeoxyuridine (Koeffler *et al.*, 1983) are devoid of inducing ability in mutant cell lines with deletions in adenosine kinase and thymidine kinase, respectively. Furthermore, induction of the erythroid differentiation of Friend cells by the aminonucleoside puromycin is inhibited by inosine (Terada *et al.*, 1978), an antagonist of this analogue nucleoside (Studzinski & Ellem, 1968). These results collectively support the concept that the activation of these analogues to the nucleotide level not only is essential for cytotoxicity but is also required for the induction of maturation.

6-Thioguanine differs from these agents in that it is an effective initiator of maturation in both Friend and HL-60 leukaemia cells deficient in hypoxanthine-guanine phosphoribosyl transferase (HGPRT) (Gusella & Houseman, 1976; Schwartz *et al.*, 1982; Gallagher *et al.*, 1984; Ishiguro *et al.*, 1984) but at best is only weakly active against parental wild type cells (Papac *et al.*, 1980; Gallagher *et al.*, 1984; Ishiguro *et al.*, 1984).

Considerable evidence is available to support the concept that 6-thioguanine, like most purines and pyrimidines and their nucleosides, must be converted to the nucleotide level to express cytotoxicity (see for example, Brockman, 1963). Multiple sites of action have been described for thioguanine nucleotides, including their incorporation into both RNA and DNA (Sartorelli *et al.*, 1958; LePage, 1960; LePage & Jones, 1961; LePage & Howard, 1963; Bieber & Sartorelli, 1964; Kwan *et al.*, 1973; Carrico & Sartorelli, 1977), and inhibition of glutamine phosphoribosylpyrophosphate amidotransferase (McCollister *et al.*, 1964; Hill & Bennett, 1969), inosine 5′-phosphate dehydrogenase (Hampton, 1963; Miech *et al.*, 1967), adenosine triphosphate: guanosine 5′-phosphate phosphotransferase (Miech *et al.*, 1969) and glycoprotein biosynthesis (Lazo *et al.*, 1977; 1979). To identify the metabolic form(s) of 6-thioguanine responsible for the induction of the differentiation of HL-60/HGPRT$^-$ cells, intracellular and extracellular metabolites of thioguanine were analyzed (Ishiguro *et al.*, 1984). The purine antimetabolite was not appreciably metabolized by these cells, indicating that 6-thioguanine itself was the metabolic form which initiated maturation. The capacity of 6-thioguanosine and $\beta$-2′-deoxythioguanosine to induce differentiation was also examined. When HL-60/HGPRT$^-$ cells were incubated with these nucleosides, large amounts of the nucleosides were converted to 6-thioguanine by purine nucleoside phosphorylase (PNP); the simultaneous exposure of the cells to 6-thioguanosine or $\beta$-2′-deoxythioguanosine and 8-aminoguanosine, an inhibitor of PNP activity, minimized degradation of the nucleosides. Under these conditions, intracellular accumulation of thioguanine nucleotides occurred, presumably through the action of nucleoside kinase activities, and no induction of differentiation was observed. Deoxycytidine kinase (DCK) has been shown to phosphorylate $\beta$-2′-deoxythioguanosine (Nakai & LePage, 1972). To eliminate the action of DCK, a double mutant of HL-60 cells deficient in both HGPRT and DCK activities was developed. In these cells, $\beta$-2′-deoxythioguanosine was devoid of cytotoxicity and was an effective inducer of maturation. The potency of deoxythioguanosine as an inducer in this sytem was not prevented by the presence of 8-aminoguanosine, suggesting that deoxythioguanosine itself was also an active initiator of maturation. The findings also suggested that the formation of 6-thioguanine nucleotides serves to antagonize the maturation process in addition to producing cytotoxicity. Furthermore, the studies demonstrate that the metabolic form(s) of the purine antimetabolite which induce differentiation differ from those that produce cytotoxicity, implying that the biochemical events required to arrest growth by these different processes are distinct. In support of this conclusion, differences occurred in the stage of cell cycle arrest produced by 6-thioguanine in parental and HL-60/HGPRT$^-$ cells (Schwartz *et al.*, 1985). Wild type HL-60 cells treated with the purine antimetabolite accumulated in the S and $G_2+M$ phases of the cell cycle, a finding similar to that occurring in other cell lines demonstrating toxicity when treated with this agent (Wotring & Roti Roti, 1980). In contrast, HL-60/HGPRT$^-$ cells which terminate proliferation through maturation when treated with 6-thioguanine accumulated in $G_1$. Arrest of cells in $G_1$ is not unexpected for mature cells (see for example, Prescott, 1976). In support of this concept, both HL-60 and HL-60/HGPRT$^-$ cells respond to dimethylsulphoxide by the initiation of differentiation and an increase in $G_1$ cells.

Parental HL-60 cells respond to the exposure to 6-thioguanine with cytotoxicity rather than differentiation; this action is due to the fact that the concentration of the purine antimetabolite required to induce maturation in this cell line is significantly higher than that required to produce cytotoxicity. Toxicity of 6-thioguanine to parental cells could be circumvented by exposing thioguanine treated HL-60 cells simultaneously to hypoxanthine or its nucleosides (Ishiguro & Sartorelli, 1985). Under these conditions, accumulation of 6-thioguanine nucleotides, the cytotoxic form(s) of this agent in HL-60 cells treated with the purine antimetabolite, was markedly decreased by the presence of the physiological purine. This resulted in a marked decrease in the cytotoxic potency of thioguanine, and the differentiation inducing properties of the purine antimetabolite were expressed. Hypoxanthine protects against the cytodestructive properties of thioguanine by preventing the anabolism of the purinethiol to its nucleotide level; this may occur through competition between hypoxanthine and 6-thioguanine at several levels. These include (a) the transport carrier for hypoxanthine/guanine (Plagemann *et al.*, 1981); (b) the enzyme HGPRT; and (c) 5-phosphoribosyl 1-pyrophosphate (PRPP), with the activation of hypoxanthine to inosine 5′-phosphate leading to a decreased availability of PRPP for the synthesis of 6-thioguanosine 5′-phosphate by HGPRT. Information of this kind would appear to be critical before an agent such as 6-thioguanine is tested for its capacity to induce maturation in a clinical setting.

### Conclusions

A few trials have been conducted to determine whether the induction of leukaemia cell differentiation could be attained in a clinical setting (see for appropriate references, Schwartz & Wiernik, 1985). These investigations suffer from an inability to differentiate between the cytotoxic and maturation producing actions of the agents employed. In addition, considerable additional fundamental information would appear to be required to most efficaciously apply the concepts of differentiation therapy to the treatment of malignant diseases of man. Phenomena such as commitment time, which often requires one to two cell generations of continuous exposure in culture to inducing agent to achieve an irreversible commitment to form end-stage mature cells, suggests that continuous infusion of agents under test would be required for this period of time for optimum effects in patients with malignant disease. This possible requirement is sorely in need of evaluation in an animal system. The phenomenon of memory, whereby cells exposed to an inducer of differentiation for less than the period of time required to achieve commitment retain this information for an additional division cycle or more, and undergo a significant degree of differentiation when cells are reexposed to inducer for a relatively short period of time consistent with an appropriate cumulative exposure to that inducer (see for example, Fibach *et al.*, 1979; Murate *et al.*, 1984; Yen *et al.*, 1984; Yen, 1984) is a potentially important phenomenon to clinical usage of agents of this kind. We have found that precommitment to memory initiated by a priming dose of one agent can be imparted to different inducing agents, making it possible to employ the clinical concept of spreading drug toxicity over several organ systems, while affecting the total neoplastic cell population. Since tumour cell heterogeneity would be expected to limit the effectiveness of a single inducer of differentiation it will be important to seek combinations of agents that act in a synergistic manner in the same cells, as well as on different portions of the cell population, to initiate maturation. Furthermore, since it is probable that all cells in a malignant cell population will not have the capacity to respond to inducers of maturation to generate end-stage cells with no proliferative capacity, it is also critical to seek mixtures of agents that would function through both differentiation and cytotoxic actions without mutual antagonism. Differentiation therapy as an approach can be envisioned to yield major decreases in the neoplastic cell burden without the degree of morbidity produced by aggressive therapy with cytodestructive agents used in combination. Effective introduction of differentiating agents into cancer therapy if attained will be an important addition to our therapeutic armamentarium.

The research from the author's laboratory described in this report was supported in part by US Public Health Service Grants CA-02817, CA-28852, and CA-08341 from the National Cancer Institute.

### References

ABE, E., MIYAURA, C., SAKAGAMI, H. & 5 others (1981). Differentiation of mouse myeloid leukemia cells induced by $1\alpha,25$-dihydroxyvitamin $D_3$. *Proc. Natl Acad. Sci.*, **78**, 4990.

BIEBER, A.L. & SARTORELLI, A.C. (1964). The metabolism of thioguanine in purine analog-resistant cells. *Cancer Res.*, **24**, 1210.

BODNER, A.J., TING, R.C. & GALLO, R.C. (1981). Induction of differentiation of human promyelocytic leukemia cells (HL-60) by nucleosides and methotrexate. *J. Natl Cancer Inst.*, **67**, 1025.

BREITMAN, T.R., SELONICK, S.E. & COLLINS, S.J. (1980). Induction of differentiation of the human promyelocytic leukemia cell line (HL-60) by retinoic acid. *Proc. Natl. Acad. Sci.*, **77**, 2936.

BRINSTER, R.L. (1974). The effects of cells transferred into the mouse blastocyst on subsequent development. *J. Exp. Med.*, **140**, 1049.

BROCKMAN, R.W. (1963). Mechanisms of resistance to anticancer agents. *Adv. Cancer Res.*, **7**, 129.

CARRICO, C.K. & SARTORELLI, A.C. (1977). Effect of 6-thioguanine on RNA biosynthesis in regenerating liver. *Cancer Res.*, **37**, 1876.

CLINE, P.R. & RICE, R.H. (1983). Modulation of involucrin and envelope competence in human keratinocytes by hydrocortisone, retinyl acetate, and growth arrest. *Cancer Res.*, **43**, 3203.

COLLINS, S.J., BODNER, A., TING, R. & GALLO, R.C. (1980). Induction of morphological and functional differentiation of human promyelocytic leukemia cells (HL-60) by compounds which induce differentiation of murine leukemia cells. *Int. J. Cancer*, **25**, 213.

COSSU, G., KUO, A.L., PESSANO, S., WARREN, L. & COOPER, R.A. (1982). Decreased synthesis of high molecular weight glycopeptides in human promyelocytic leukemia cells (HL-60) during phorbol ester-induced macrophage differentiation. *Cancer Res.*, **42**, 484.

COWLEY, G., SMITH, J.A., GUSTERSON, B., HENDLER, F. & OZANNE, B. (1984). The amount of EGF receptors is elevated on squamous cell carcinomas. In *Cancer Cells 1. The Transformed Phenotype*. (Eds. Levine *et al.*) p. 5. Cold Spring Harbor: New York.

CREEK, K.E., MORRÉ, J.D., SILVERMAN-JONES, C.S., SHIDOJI, Y. & DELUCA, L.M. (1983). Mannosyl carrier functions of retinyl phosphate and dolichyl phosphate in rat liver endoplasmic reticulum. *Biochem. J.*, **210**, 541.

CROOKE, S.T., DUVERNAY, V.H., GALVAN, L. & PRESTAYKO, A.W. (1978). Structure-activity relationships of anthracyclines relative to effects on macromolecular syntheses. *Mol. Pharmacol.*, **14**, 290.

DECOSSE, J.J., GOSSENS, C.L., KUZMA, J.F. & UNSWORTH, B.R. (1973). Breast cancer: Induction of differentiation by embryonic tissue. *Science*, **181**, 1057.

DEJAGER, R., DELGADO, M., BAYSSAS, M. & 5 others (1981). Phase II study of aclacinomycin in acute leukemia and leukemic lymphosarcoma. *Proc. Am. Assoc. Cancer Res.*, **22**, 171 (Abstract).

DUVERNAY, V.H., ESSERY, J.M., DOYLE, T.W., BRADNER, W.T. & CROOKE, S.T. (1979*a*). The antitumor effects of anthracyclines. The importance of the carbomethoxy-group at position-10 of marcellomycin and rudolfomycin. *Mol. Pharmacol.*, **15**, 341.

DUVERNAY, V.H., PACHTER, J.A. & CROOKE, S.T (1979*b*). Deoxyribonucleic acid binding studies on several new anthracycline antitumor antibiotics. Sequence preference and structure-activity relationships of marcellomycin and its analogues as compared to adriamycin. *Biochemsitry*, **18**, 4024.

EBERT, P.S., WARS, I. & BUELL, D.N. (1976). Erythroid differentiation in cultured Friend leukemia cells treated with metabolic inhibitors. *Cancer Res.*, **36**, 1809.

FALCONE, G., TATÔ, F. & ALEMÀ, S. (1985). Distinctive effects of the viral oncogenes *myc*, *erb*, *fps*, and *src* on the differentiation program of quail myogenic cells. *Proc. Natl Acad. Sci.*, **82**, 426.

FELSTED, R.L., GUPTA, S.K., GLOVER, C.J., FISCHKOFF, S.A. & GALLAGHER, R.E. (1983). Cell surface membrane protein changes during the differentiation of cultured human promyelocytic leukemia HL-60 cells. *Cancer Res.*, **43**, 2754.

FIBACH, E., GAMBERI, R., SHAW, P.A. & 5 others (1979). Tumor promoter-mediated inhibition of cell differentiation: Suppression of the expression of erythroid functions in murine erythroleukemia cells. *Proc. Natl Acad. Sci.*, **76**, 1906.

FRIEND, C., SCHER, W., HOLLAND, J.G. & SATO, T. (1971). Hemoglobin synthesis in murine virus-induced leukemia cells *in vitro*: Stimulation of erythroid differentiation by dimethyl sulfoxide. *Proc. Natl Acad. Sci.*, **68**, 378.

FUCHS, E. & GREEN, H. (1981). Regulation of terminal differentiation of cultured human keratinocytes by vitamin A. *Cell*, **25**, 617.

GALLAGHER, R.E., FERRARI, A.C., ZULICH, A.W., YEN, R-W.-C. & TESTA, J.R. (1984). Cytotoxic and cyto-differentiative components of 6-thioguanine resistance in HL-60 cells containing acquired double minute chromosomes. *Cancer Res.*, **44**, 2642.

GALLO, R.C., BREITMAN, T.R. & RUSCETTI, F.W. (1982). Proliferation and differentiation of human myeloid leukemia cell lines *in vitro*. In: *Maturation Factors and Cancer* (Ed. Moore), p. 255. Raven Press, New York.

GARRET, C. & KREDICH, N.M. (1981). Induction of hemoglobin synthesis by xylosyladenine in murine erytholeukemia cells. Metabolism of xylosyladenine and effects on transmethylation. *J. Biol. Chem.*, **256**, 12705.

GOOTWINE, E., WEBB, C. & SACHS, L. (1982). Participation of myeloid leukemic cells injected into embryos on hematopoietic differentiation in adult mice. *Nature*, **299**, 63.

GREEN, H. & WATT, F.M. (1982). Regulation by vitamin A of envelope cross-linking in cultured keratinocytes derived from different human epithelia. *Mol. Cell. Biol.*, **2**, 1115.

GUSELLA, J., GELLER, R., CLARKE, B., WEEKS, V. & HOUSEMAN, D. (1976). Commitment to erythroid differentiation by Fried erythroleukemia cells: A stochastic analysis. *Cell*, **9**, 221.

GUSELLA, J.F. & HOUSEMAN, D. (1976). Induction of erythroid differentiation *in vitro* by purines and purine analogues. *Cell*, **8**, 263.

HAMPTON, A. (1963). Reactions of ribonucleotide derivatives of purine analogues at the catalytic site of inosine 5′-phosphate dehydrogenase. *J. Biol. Chem.*, **238**, 3068.

HEIFETZ, A., KEENAN, R.W. & ELBEIN, A.D. (1979). Mechanism of action of tunicamycin on the UDP-glcNAc: Dolichylphosphate glcNAc-1-phosphate transferase. *Biochemistry*, **8**, 2186.

HILL, D.L. & BENNETT, L.L., Jr. (1969). Purification and properties of 5-phosphoribosylpyrophosphate amido-transferase from adenocarcinoma 755 cells. *Biochemistry*, **8**, 122.

HOLBROOK, K.A. & HENNINGS, H. (1983). Phenotypic expression of epidermal cells *in vitro*: A review. *J. Invest. Derm.*, **81**, 11s.

HUBERMAN, E. & CALLAHAM, M.F. (1979). Induction of terminal differentiation in human promyelocytic leukemia cells by tumor-promoting agents. *Proc. Natl Acad. Sci.*, **76**, 1293.

ISHIGURO, K. & SARTORELLI, A.C. (1985). Enhancement of the differentiation-inducing properties of 6-thioguanine by hypoxanthine and its nucleosides in HL-60 promyelocytic leukemia cells. *Cancer Res.*, **45**, 91.

ISHIGURO, K., SCHWARTZ, E.L. & SARTORELLI, A.C. (1984). Characterization of the metabolic forms of 6-thioguanine responsible for cytotoxicity and induction of differentiation of HL-60 acute promyelocytic leukemia cells. *J. Cell Physiol.*, **121**, 383.

KIM, K.H., SCHWARTZ, F. & FUCHS, E. (1984). Differences in keratin synthesis between normal epithelial cells and squamous cell carcinomas are mediated by vitamin A. *Proc. Natl Acad. Sci.*, **81**, 4280.

KING, C.L. & SARTORELLI, A.C. (1985). Oncogene expression and the role of epidermal growth factor receptor (EGFR) in the terminal differentiation of cultured human malignant keratinocytes (SqCC/Y1). *Proc. Am. Assoc. Cancer Res.*, **26**, 40 (Abstract).

KOEFFLER, H.P., YEN, J. & CARLSON, J. (1983). The study of human myeloid differentiation using bromodeoxyuridine (BrdU). *J. Cell. Physiol.*, **116**, 111.

KWAN, S.-W., KWAN, S.-P. & MANDEL, H.G. (1973). The incorporation of 6-thioguanine into RNA fraction and its effect on RNA and protein biosynthesis in mouse sarcoma 180 ascites cells. *Cancer Res.*, **33**, 950.

LAZO, J.S., HWANG, K.M. & SARTORELLI, A.C. (1977). Inhibition of L-fucose incorporation into glycoprotein of sarcoma 180 cells by 6-thioguanine. *Cancer Res.*, **37**, 4250.

LAZO, J.S., SHANSKY, C.W. & SARTORELLI, A.C. (1979). Reduction in cell surface concanavalin A binding and mannose incorporation into glycoproteins of sarcoma 180 by 6-thioguanine. *Biochem. Pharmacol.*, **28**, 583.

LePAGE, G.A. (1960). Incorporation of 6-thioguanine into nucleic acids. *Cancer Res.*, **20**, 403.

LePAGE, G.A. & HOWARD, N. (1963). Chemotherapy studies on mammary tumors of C3H mice. *Cancer Res.*, **23**, 622.

LePAGE, G.A. & JONES, M. (1961). Further studies on mechanism of action of 6-thioguanine. *Cancer Res.*, **21**, 1590.

LOTEM, J. & SACHS, L. (1974). Different blocks in the differentiation of myeloid leukemic cells. *Proc. Natl Acad. Sci.*, **71**, 3507.

LOTEM, J. & SACHS, L. (1975). Induction of specific changes in the surface membrane of myeloid leukemic cells by steroid hormones. *Int. J. Cancer*, **15**, 731.

LOTEM, J. & SACHS, L. (1979). Regulation of normal differentiation in mouse and human myeloid leukemia cells by phorbol esters and the mechanism of tumor promotion. *Proc. Natl Acad. Sci.*, **76**, 5158.

LUCAS, D.L., ROBINS, R.K., KNIGHT, R.D. & WRIGHT, D.G. (1983*a*). Induced maturation of the human promyelocytic leukemia cell line, HL-60, by 2-β-D-ribofuranosylselenazole-4-carboxamide. *Biochem. Biophys. Res. Commun.*, **115**, 971.

LUCAS, D.L., WEBSTER, H.K. & WRIGHT, D.G. (1983*b*). Purine metabolism in myeloid precursor cells during maturation. Studies with the HL-60 cell line. *J. Clin. Invest.*, **72**, 1889.

McCOLLISTER, R.J., GILBERT, W.R., Jr., ASHTON, D.W. & WYNGAARDEN, J.B. (1964). Pseudofeedback inhibition of purine synthesis by 6-mercaptopurine ribonucleotide and other purine analogues. *J. Biol. Chem.*, **239**, 1560.

METCALF, D., MOORE, M.A.S. & WARNER, N.L. (1969). Colony formation *in vitro* by myelomonocytic leukemic cells. *J. Natl Cancer Inst.*, **43**, 983.

MIECH, R.P., PARKS, R.E., Jr., ANDERSON, J.H., Jr. & SARTORELLI, A.C. (1967). An hypothesis on the mechanism of action of 6-thioguanine. *Biochem. Pharmacol.*, **16**, 2222.

MIECH, R.P., YORK, R. & PARKS, R.E., Jr. (1969). Adenosine triphosphate-guanosine 5′-phosphate phosphotransferase. II. Inhibition by 6-thioguanosine 5′-phosphate of the enzyme isolated from hog brain and sarcoma 180 ascites cells. *Mol. Pharmacol.*, **5**, 30.

MORIN, M.J. & SARTORELLI, A.C. (1984). Inhibition of glycoprotein biosynthesis by the inducers of HL-60 cell differentiation, aclacinomycin A and marcellomycin. *Cancer Res.*, **44**, 2807.

MURATE, T., KANEDA, T., RIFKIND, R.A. & MARKS, P.A. (1984). Inducer-mediated commitment of murine erythroleukemia cells to terminal cell division: The expression of commitment. *Proc. Natl Acad. Sci.*, **81**, 3394.

NAKAI, Y. & LePAGE, G.A. (1972). Characterization of the kinase(s) involved in the phosphorylation of α- and β-2′-deoxythioguanosine. *Cancer Res.*, **32**, 2445.

NAKAYASU, M., TERDA, M., TAMURA, G. & SUGIMURA, T. (1980). Induction of differentiation of human and murine myeloid leukemia cells in culture by tunicamycin. *Proc. Natl Acad. Sci.*, **77**, 409.

OMARY, M.B. & TROWBRIDGE, I.S. (1981). Biosynthesis of the human transferrin receptor in cultured cells. *J. Biol. Chem.*, **256**, 12888.

OSBORNE, H.B., BAKKE, A.C. & YU, J. (1982). Effect of dexamethasone on hexamethylene bisacetamide-induced Friend cell erythrodifferentiation. *Cancer Res.*, **42**, 513.

PAINTER, R.B. (1978). Inhibition of DNA replicon initiation by 4-nitroquinoline 1-oxide, adriamycin, and ethyleneimine. *Cancer Res.*, **38**, 4445.

PAPAC, R.J., BROWN, A.E., SCHWARTZ, E.L. & SARTORELLI, A.C. (1980). Differentiation of human promyelocytic leukemia cells *in vitro* by 6-thioguanine. *Cancer Lett.*, **10**, 33.

PARAN, M., SACHS, L., BARAK, Y. & RESNITZKY, P. (1970). *In vitro* induction of granulocytic differentiation in hematopoietic cells from leukemic and non-leukemic patients. *Proc. Natl Acad. Sci.*, **67**, 1542.

PARKINSON, E.K., GRABHAM, P. & EMMERSON, A. (1983). A subpopulation of cultured keratinocytes which is resistant to the induction of terminal differentiation-related changes by phorbol, 12-myristate, 13-acetate: Evidence for an increase in the resistant population following transformation. *Carcinogenesis*, **4**, 857.

PIERCE, G.B., NAKANE, P.K., MARTINEZ-HERMANDEZ, A. & WARD, J.M. (1977). Ultrastructural comparison of differentiation of stem cells of murine adenocarcinomas of colon and breast with their normal counterparts. *J. Natl Cancer Inst.*, **58**, 1329.

PIERCE, G.B. & WALLACE, C. (1971). Differentiation of malignant to benign cells. *Cancer Res.*, **31**, 127.

PLAGEMANN, P.G.W., MARZ, R., WOHLHUETER, R.M., GRAFF, J.C. & ZYLKA, J.M. (1981). Facilitated transport of 6-mercaptopurine and 6-thioguanine and non-mediated permeation of 8-azaguanine in Novikoff rat hepatoma cells and relationship to intracellular phosphorylation. *Biochim. Biophys. Acta*, **647**, 49.

PRASAD, K.N. (1983). Therapeutic potentials of differentiating agents in neuroblastomas. *Prog. Clin. Biol. Res.*, **132C**, 75.

PRESCOTT, D.M. (1976). Cell cycle and control of cellular reproduction. *Adv. Genet.*, **18**, 99.

REISS, M., PITMAN, S.W. & SARTORELLI, A.C. (1985). Modulation of the terminal differentiation of human squamous carcinoma cells *in vitro* by all-trans retinoic acid. *J. Natl Cancer Inst.*, **74**, 1015.

REUBEN, R.C., RIFKIN, R.A. & MARKS, P.A. (1980). Chemically induced murine erythroleukemia differentiation. *Biochim. Biophys. Acta*, **605**, 325.

RHEINWALD, J.G. & BECKETT, M.A. (1980). Defective terminal differentiation in culture as a consistent and selectable character of malignant human keratinocytes. *Cell*, **22**, 629.

RICE, R.H., CLINE, P.R. & COE, E.L. (1983). Mutually antagonistic effects of hydrocortisone and retinyl acetate on envelope competence in cultured malignant human keratinocytes. *J. Invest. Derm.*, **81**, 176s.

SAKURAI, M., SAMPI, K. & HOZUMI, H. (1983). Possible differentiation of human acute myeloblastic leukemia cells by daily and intermittent administration of aclacinomycin A. *Leuk. Res.*, **7**, 139.

SARTORELLI, A.C., LEPAGE, G.A. & MOORE, E.C. (1958). Metabolic effects of 6-thioguanine. I. Studies on thioguanine-resistant and -sensitive Ehrlich ascites cells. *Cancer Res.*, **18**, 1232.

SCHER, W. & FRIEND, C. (1978). Breakage of DNA and alteration in folded genomes by inducers of differentiation in Friend erythroleukemia cells. *Cancer Res.*, **38**, 841.

SCHUBERT, D., HUMPHREYS, S., JACOB, F. & DEVITRY, F. (1971). Induced differentiation of a neuroblastoma. *Dev. Biol.*, **25**, 514.

SCHULMAN, H.M., WILCZYNSKA, A. & PONKA, P. (1981). Transferrin and iron uptake by human lymphoblastoid and K-562 cells. *Biochem. Biophys. Res. Commun.*, **100**, 1523.

SCHWARTZ, E.L., BROWN, B.J., NIERENBURG, M., MARSH, J.C. & SARTORELLI, A.C. (1983*a*). Evaluation of some anthracycline antibiotics in an *in vivo* model for studying drug-induced human leukemia cell differentiation. *Cancer Res.*, **43**, 2725.

SCHWARTZ, E.L., ISHIGURO, K. & SARTORELLI, A.C. (1983*b*). Induction of leukemia cell differentiation by chemotherapeutic agents. *Adv. Enz. Reg.*, **21**, 3.

SCHWARTZ, E.L. & SARTORELLI, A.C. (1982). Structure-activity relationships for the induction of differentiation of HL-60 human acute leukemia cells by anthracyclines. *Cancer Res.*, **42**, 2651.

SCHWARTZ, E.L., SNODDY, J.R., KREUTTER, D., RASMUSSEN, H. & SARTORELLI, A.C. (1983*c*). Synergistic induction of HL-60 differentiation by 1,25-dihydroxyvitamin $D_3$ and dimethyl sulfoxide (DMSO). *Proc. Am. Assoc. Cancer Res.*, **24**, 18 (Abstract).

SCHWARTZ, E.L. & WIERNIK, P.H. (1985). Differentiation of leukemia cells by chemotherapeutic agents. *Leuk Res.* (in press).

SKUBITZ, K.M. & AUGUST, J.T. (1983). Analysis of cell-surface protein changes accompanying differentiation of HL-60 cells. *Arch. Biochem. Biophys.*, **226**, 1.

SOKOLSKI, J.A. & SARTORELLI, A.C. (1984). Induction of differentiation of HL-60 promyelocytic leukemia cells by inhibitors of IMP dehydrogenase. *Proc. Am. Assoc. Cancer Res.*, **25**, 42 (Abstract).

STUDZINSKI, G.P. & ELLEM, K.A.O. (1968). Differences between diploid and heteroploid cultured mammalian cells in their response to puromycin aminonucleoside. *Cancer Res.*, **28**, 1773.

SUN, T.T. & GREEN, H. (1976). Differentiation of the epidermal keratinocyte in cell culture: Formation of the cornified envelope. *Cell*, **9**, 511.

TAETLE, R., HONEYSETT, J.M. & TROWBRIDGE, I.S. (1983). Effects of anti-transferrin receptor antibodies on growth of normal and malignant myeloid cells. *Int. J. Cancer*, **32**, 343.

TAETLE, R., RHYNER, K., CASTAGNOLA, J., TO, D. & MENDELSOHN, J. (1985). Role of transferrin, Fe, and transferrin receptors in myeloid leukemia cell growth. *J. Clin. Invest.*, **75**, 1061.

TAKENAGA, K., HOZUMI, M. & SAKAGAMI, Y. (1980). Effects of retinoids on induction of differentiation of cultured mouse myeloid leukemia cells. *Cancer Res.*, **40**, 914.

TEI, D., MAKINO, Y., SAKAGAMI, H., KANAMARA, I. & KONNO, K. (1982). Decrease of transferrin receptor during mouse myeloid leukemia (M1) cell differentiation. *Biochem. Biophys. Res. Commun.*, **107**, 1419.

TERADA, M., EPNER, E., NUDEL, U. & 4 others. (1978). Induction of murine erythroleukemia differentiation by actinomycin D. *Proc. Natl Acad. Sci.*, **75**, 2795.

TESTA, U., THOMOPOULOS, P., VINCI, G. & 4 others (1982). Transferrin binding to K562 cell line. Effect of heme and sodium butyrate induction. *Exp. Cell Res.*, **140**, 251.

TROWBRIDGE, I.S., LESLEY, J. & SCHULTE, R. (1982). Murine cell surface transferrin receptor: Studies with an anti-receptor monoclonal antibody. *J. Cell. Physiol.*, **112**, 403.

TROWBRIDGE, I.S. & LOPEZ, F. (1982). Monoclonal antibody to transferrin receptor blocks transferrin binding and inhibits human tumor cell growth *in vitro*. *Proc. Natl Acad. Sci.*, **79**, 1175.

WARRELL, R.P., ARLIN, Z., GEE, T., LACHER, M. & YOUNG, C. (1981). Phase I-II evaluation of aclacinomycin A in acute leukemia. *Proc. Am. Assoc. Cancer Res.*, **22**, 191 (Abstract).

WILLIE, J.J., Jr., PITTLEKOW, M.R., SHIPLEY, G.D. & SCOTT, R.E. (1984). Integrated control of growth and differentiation of normal human prokeratinocytes cultured in serum-free medium: Clonal analyses, growth kinetics, and cell cycle studies. *J. Cell. Physiol.*, **121**, 31.

WOTRING, L.L. & ROTI ROTI, J.L. (1980). Thioguanine-induced S and $G_2$ blocks and their significance to the mechanism of cytotoxicity. *Cancer Res.*, **40**, 1458.

YAMADA, K., NAKAMURA, T., TSURAO, T. & 14 others (1980). A phase II study of aclacinomycin A in acute leukemia in adults. *Cancer Treat. Rep.*, **7**, 177.

YEH, C.G., PAPAMICHAEL, M. & FAULK, W.P. (1982). Loss of transferrin receptors following induced differentiation of HL-60 promyelocytic leukemia cells. *Exp. Cell Res.*, **138**, 429.

YEN, A. (1984). Control of HL-60 myeloid differentiation. Evidence of uncoupled growth and differentiation control, S-phase specificity, and two-step regulation. *Exp. Cell Res.*, **156**, 198.

YEN, A., REECE, S.A. & ALBRIGHT, K.L. (1984). Dependence of HL-60 myeloid cell differentiation on continuous and split retinoic acid exposures: Precommitment memory associated with altered nuclear structures. *J. Cell. Physiol.*, **118**, 277.

YOUNG, R.C., OZOLS, R.F. & MYERS, C.E. (1981). The anthracycline antineoplastic drugs. *N. Engl. J. Med.*, **305**, 139.

YUSPA, S.H. & MORGAN, D.L. (1981). Mouse skin cells resistant to terminal differentiation associated with initiation of carcinogenesis. *Nature*, **293**, 72.